# Challenges in targeting the tryptophan metabolism in cancer immunotherapy

**DOI:** 10.18632/aging.203492

**Published:** 2021-08-26

**Authors:** Julia Schollbach, Stefan Löb

**Affiliations:** 1 Department of General, Visceral, Transplantation, Vascular and Pediatric Surgery, University Hospital Würzburg, Würzburg, Germany

**Keywords:** cancer, immumnotherapy, indoleamine-2,3-dioxygenase 1, tryptophan catabolism, clinical trials

In the past few decades, Indoleamine 2,3-dioxygenase 1 (IDO1) - as the rate-limiting enzyme in the metabolism of the essential amino acid tryptophan – has been considered as a promising target in the expanding field of cancer immunotherapy. Recent failure of a phase III study with a IDO1 inhibitor initially dampened this euphoria. However, this might be seen as a chance for the development of novel IDO1-targeted therapeutic strategies in cancer therapy, because the exact role of IDO1 in the tumor environment remains still complex and not fully understood.

It has been over 20 years since Munn and Mellor identified the tryptophan-catabolizing enzyme indoleamine-2,3-dioxygenase-1 (IDO1) as a key mechanism of T-cell-mediated immunosuppression in a pioneering study in pregnant mice [1]. The concept of IDO1-mediated immune regulation (i.e. suppression of local CD8+ T effector cells and natural killer cells and induction of CD4+ T regulatory cells induced by tryptophan depletion and by accumulating immunosuppressive metabolites of the kynurenine pathway) was quickly extended to malignant diseases in a variety of studies [2].

Expression of IDO1 was extensively studied in different tumor tissues [2] and overexpression of IDO1 was often correlated with a less favorable prognosis in several types of human cancer, although the causal relationship has not yet been conclusively explained by these correlative observations. Inconsistencies appear when studies are compared with regard to the expression profiles of IDO1 (constitutive vs. inducible by inflammatory stimuli), the nature of IDO1 expressing cells (tumor cells vs. antigen-presenting cells), the density of IDO1 expression and its localization within different tumor compartments (tumor centre vs. invasive tumor front). Data remain heterogeneous and we still don’t really know if IDO1 is a friend or foe in terms of tumor immune escape [3].

However, inhibition of IDO1 has attracted enormous attention in the fast growing area of immunooncology in cancer therapy. Several orally available IDO1-inhibitors such as Epacadostat and 1-D-Methyl-Tryptophan (1-D-MT, Indoximod) have entered human clinical trials over the last few years without a major safety concern – although we are far away from fully understanding the function of Epacadostat and 1-D-MT *in vivo* [4]. Furthermore, we and others have shown that 1-D-MT does not block the activity of IDO1 activity in human cancers [5]. Taken together, 1-D-MT might exert any antitumor effects via off-target mechanisms in terms if IDO1 inhibition or via alternative targets rather than IDO1. Epacadostat, a more potent and selective IDO1 inhibitor, increased T and NK cell proliferation as well as IFN-γ production and suppressed regulatory T cells *in vitro*. However, in vivo, Epacadostat showed antitumor activity only in immunocompetent but not in immunodeficient or IDO1 knockout mouse models [6].

In the past, the use of the two predominant IDO1 inhibitors Indoximod and Epacadostat as monoagents produced rather manageable clinical results (for example, it has not been shown that IDO1 inhibitors have any single-agent activity in the treatment of melanoma patients). Therefore, further studies concentrated on the combination of IDO1 inhibition with established chemotherapeutic agents. Checkpoint inhibitors such as programmed cell death protein 1 (PD1) inhibitors (pembrolizumab, nivolumab), cytotoxic T lymphocyte antigen 4 (CTLA-4) antibodies (ipilimumab) or programmed death ligand 1 (PD-L1) antibodies (including atezolizumab) turned out as interesting combination partners.

Surprisingly, the latest results of the ECHO-301 study (the first double-blind phase III study in several solid human tumors) showed that Epacadostat failed to achieve improved disease-free survival in addition to anti-PD1 immune checkpoint inhibitor therapy compared to Pembrolizumab alone [7]. As a consequence, other trials of different IDO inhibitors have been scaled back or even halted.

The reasons for failure seem to be complex: jumping directly from unselected small phase I/II trials to a large, randomized phase III trial, insufficient inhibition of the target due to low dosage of Epacadostat with no pharmacokinetic and pharmacodynamic measurement in patient sera, an unspecific selection of IDO1-expressing tumors and the unknown role of other enzymes along the kynurenine pathway in human tumors like IDO2 and TDO (tryptophan 2,3-dioxygenase) [8].

So, what’s next after this setback in the development of IDO1 inhibitors? Targeting the tryptophan metabolism in human tumors still offers promise. IDO1 inhibitors have been investigated in a lot of preclinical and clinical studies and seem to have an additive and synergistic effect with established chemotherapeutic agents. But there is definitely more research to be done to understand the functional role of IDO1 within the tumor microenvironment and to collect more knowledge on the exact effect of IDO1 inhibition before heading out to the next large-scale phase III study. In addition, more precise investigations appear to be required in order to identify patient subgroups who could benefit from IDO1 inhibition in the context of already established neoadjuvant and adjuvant therapy strategies.

Taking a backward step in studying IDO1-mediated tumor immune escape may help us to start a steep take-off in this exciting field of translational immunotherapy.
